# Hydrated lime promoted the polysaccharide content and affected the transcriptomes of *Lentinula edodes* during brown film formation

**DOI:** 10.3389/fmicb.2023.1290180

**Published:** 2023-12-04

**Authors:** Yan Li, Hongcheng Wang, Ying Zhang, Quanju Xiang, Qiang Chen, Xiumei Yu, Lingzi Zhang, Weihong Peng, Petri Penttinen, Yunfu Gu

**Affiliations:** ^1^Department of Microbiology, College of Resources, Sichuan Agricultural University, Chengdu, China; ^2^Sichuan Academy of Agricultural Sciences, Chengdu, China

**Keywords:** RNA-seq, *Lentinula edodes*, brown film, hydrated lime, CAZymes

## Abstract

Brown film formation, a unique developmental stage in the life cycle of *Lentinula edodes*, is essential for the subsequent development of fruiting bodies in *L. edodes* cultivation. The pH of mushroom growth substrates are usually adjusted with hydrated lime, yet the effects of hydrated lime on cultivating *L. edodes* and the molecular mechanisms associated with the effects have not been studied systemically. We cultivated *L. edodes* on substrates supplemented with 0% (CK), 1% (T1), 3% (T2), and 5% (T3) hydrated lime (Ca (OH)_2_), and applied transcriptomics and qRT-PCR to study gene expression on the brown film formation stage. Hydrated lime increased polysaccharide contents in *L. edodes*, especially in T2, where the 5.3% polysaccharide content was approximately 1.5 times higher than in the CK. The addition of hydrated lime in the substrate promoted laccase, lignin peroxidase and manganese peroxidase activities, implying that hydrated lime improved the ability of *L. edodes* to decompose lignin and provide nutrition for its growth and development. Among the annotated 9,913 genes, compared to the control, 47 genes were up-regulated and 52 genes down-regulated in T1; 73 genes were up-regulated and 44 were down-regulated in T2; and 125 genes were up-regulated and 65 genes were down-regulated in T3. Differentially expressed genes (DEGs) were enriched in the amino acid metabolism, lipid metabolism and carbohydrate metabolism related pathways. The carbohydrate-active enzyme genes up-regulated in the hydrated lime treatments were mostly glycosyl hydrolase genes. The results will facilitate future optimization of *L. edodes* cultivation techniques and possibly shortening the production cycle.

## 1 Introduction

*Lentinula edodes*, an edible mushroom, is among the most widely consumed mushrooms (Zhang et al., 2011). The production cycle of *Lentinula edodes* is long, including mycelium growth period, brown film formation period, primordium formation period and fruiting body development period. After maturity, mushroom mycelium forms brown film through pigment accumulation. The brown film stage occupies an important position and is an important link of nutrient accumulation and related gene expression, directly affecting the quantity and quality of fruiting bodies (Yan et al., 2020).

The cultivation of edible fungi is affected by environmental factors, and the pH of cultivation substrate is one of the most important factors (Yadav and Chandra, 2014). Sawdust is the main component of cultivation substrates. Wood degrading brown-rot fungi produce large amount of organic acids (Arantes et al., 2009; Hastrup et al., 2012), which may acidify improperly stored sawdust. Since acidified sawdust should not be cultivated directly, hydrated lime that can neutralize organic acids is often added to the substrate to regulate pH (Li et al., 2022). Proper amounts of hydrated lime in the growth substrate can also inhibit the growth of molds. Two percent hydrated lime in the growth substrate promoted the growth of *Pleurotus ostreatus* mycelia and increased its yield (Khan et al., 2013).

The formation of brown film and fruiting bodies of *L. edodes* requires the accumulation of nutrients. Carbohydrate-active enzymes (CAZymes) that degrade plant cell walls provide carbohydrates for *L. edodes* (Zhang et al., 2016). Glycoside hydrolases (GHs) hydrolyze glycosidic bonds in carbohydrates, both those between sugar moieties and between sugar and other moieties (Sathya and Khan, 2015), providing nutrition for the brown film formation of *L. edodes*. β-1,3-glucanase in the GH5 family participated in the development of fruiting bodies of *L. edodes* (Yan et al., 2021). Laccases in the auxiliary activity (AAs) category degrade lignin in the substrate and play an important role in the differentiation of the primordium into the fruiting body (Chen et al., 2003; Xie et al., 2018). Cellulase and hemicellulose, also classified as AAs, degrade cellulose and hemicellulose in substrates (Bano et al., 1993). Glycosyl transferases (GTs) catalyze glycosidic bond formation and participate in polysaccharide synthesis in mushrooms (Liang et al., 2022).

In recent years, the focus of omics analyses of *L. edodes* has mostly been on the development from mycelium to fruiting bodies (Sakamoto et al., 2017; Wang et al., 2018). To our knowledge, transcriptomics has not been applied to study how hydrated lime promotes the growth and development of *L. edodes*. To provide data for optimizing the cultivation techniques of *L. edodes*, we studied the molecular mechanisms behind the effects of hydrated lime on the growth and development of *L. edodes*. We applied RNA-seq technique to compare the transcriptomes of *L. edodes* cultivated on substrates with 0% to 5% hydrated lime. Analyzing the differences in transcription and expression of CAZymes genes in *L. edodes* under different hydrated lime concentrations and determining the optimal hydrated lime dosage will help optimizing *L. edodes* cultivation techniques.

## 2 Materials and methods

### 2.1 *Lentinula edodes* cultivation

*Lentinula edodes* ACCC50302 was obtained from the Agricultural Culture Collection of China. The synthetic substrate, consisting of 80% *Quercus acutissima* sawdust, 19% wheat bran and 1% sucrose, was used as such in the control treatment (CK) or supplemented with 1% (T1), 3% (T2), or 5% (T3) hydrated lime (Ca (OH)_2_) that was suspended into water 1:1.5 (w:vol). Mushroom culture packages with 800 g of the substrate (60% water content) were sterilized, cooled to room temperature, and inoculated with *L. edodes*. Packages were kept at 20–25°C with 50–60% relative humidity in the dark. After mycelium had fully colonized the medium, the packages were kept at 20–28°C with relative humidity below 70%. When the brown film was fully formed, the packages were transferred to a mushroom shed for fruiting body formation. The treatments consisted of three replicates with 20 packages per replicate. Part of the mycelia was collected at the brown film formation stage. The collected mycelia were mixed, frozen immediately in liquid nitrogen and stored at -80°C for extraction of RNA and enzyme activity assay. The fruiting body samples were collected at maturity, dried at 55°C, crushed and passed through a 80 mesh filter. Polysaccharides in fruiting bodies were extracted using hot water extraction and quantified using the phenol-sulfuric acid method (Wang et al., 2014).

### 2.2 Enzyme assay

Extracellular enzymes were extracted from 10 g wet weight growth substrate by suspending in 200 mL of 50 mM sodium acetate buffer (pH 4.8) and centrifuging at 180 rpm for 1 h at 4°C. The activities of lignocellulose degrading enzymes laccase, cellulase, hemicellulase, manganese peroxidase (MnP), and lignin peroxidase (LiP) in the supernatant were determined as previously described (Arora et al., 2002; Yeo et al., 2007; Prior and Day, 2008).

### 2.3 RNA Extraction, cDNA library construction and sequencing

Total RNA was isolated using the Trizol Reagent (Thermo Fisher Scientific, Franklin, MA, United States), after which the concentration and quality of the RNA were determined using a NanoDrop spectrophotometer (Thermo Fisher Scientific). The integrity of the RNA in the extracts was confirmed by electrophoresis in 1% agarose gel.

Sequencing libraries were generated from three micrograms of RNA using a TruSeq RNA Sample Preparation Kit (Illumina, San Diego, CA, United States) (Perina et al., 2017). The mRNA was purified from total RNA using poly-T-oligo-attached magnetic beads (Ozsolak and Milos, 2011). cDNA was synthesized using fragmented mRNA as template. cDNA fragments of about 200 bp were selected with an AMPure XP system (Beckman Coulter, Beverly, CA, USA). The fragments were selectively enriched with PCR, the PCR products were purified using AMPure XP system and quantified using an Agilent high sensitivity DNA assay on a Bioanalyzer 2100 system (Agilent, Waldbronn, Germany). The libraries were sequenced on a Hiseq platform (Illumina) at Shanghai Personal Biotechnology Co., Ltd (Shanghai, China). The raw sequence data were submitted to NCBI Sequence Read Archive with accession number PRJNA979791.

### 2.4 Expression analysis

Sequences were trimmed with Cutadapt v1.18 to remove 3-end adapter, and filtered to remove reads with average quality below Q20. The filtered reads were aligned to the *L. edodes* reference genome (*Lentinula_edodes*.Lened_assembly 01.dna.toplevel.fa) using HISAT2 (Kim et al., 2015). Reads per gene were counted using HTSeq v.0.9.1 (Anders et al., 2015). Expression levels were normalized by calculating fragments per kilobase of exon per million fragments mapped (FPKM). In the reference transcriptome, the genes with FPKM > 1 were considered to be expressed. Differential gene expression analysis was done using R package DEseq2 v1.8.3; genes with | log2FoldChange| > 1 and *P* < 0.05 were regarded as differentially expressed genes (DEGs) (Wang et al., 2010; Varet et al., 2016).

### 2.5 Enrichment analysis of DEGs

In the gene ontology (GO) enrichment analysis, the DEGs were mapped to GO categories using topGO and the GO database (Alexa et al., 2006). In the pathway analysis, the DEGs were mapped into Kyoto Encyclopedia of Genes and Genomes (KEGG) pathways using R package clusterprofiler v4.6.0 (Yu et al., 2012).

### 2.6 CAZymes analysis

The genes related to carbohydrate-active enzymes (CAZymes) that were differently expressed in the transcriptome of *L. edodes* were analyzed using the CAZymes Analysis Toolkit (CAT) and the carbohydrate enzyme database^1^ (Cantarel et al., 2009; Park et al., 2010).

### 2.7 Quantitative real-time PCR

Six differentially expressed CAZymes genes were chosen for validating the expression based on their biological roles in GO and KEGG analysis and the differences in gene expression between samples. The real-time PCR (qRT-PCR) was performed on a CFX96 Real-Time System (BIO-RAD) according to the manufacturer’s instructions with glyceraldehyde-3-phosphate dehydrogenase (GAPDH) gene as the internal control gene and three biological replicates with three technical replicates. cDNA samples from the same batch of sequencing were used as templates. The primers used are in Supplementary Table 1.

### 2.8 Statistical analysis

Differences between treatments were tested using one-way ANOVA and Tukey’s test in IBM SPSS Statistics for Windows V27.0 (IBM Corp., Armonk, NY, USA). GraphPad Prism V9.4.1 for Windows (GraphPad Software, San Diego, California, USA) was used for fitting and plotting.

## 3 Results

### 3.1 Phenotypic traits

**FIGURE 1:**
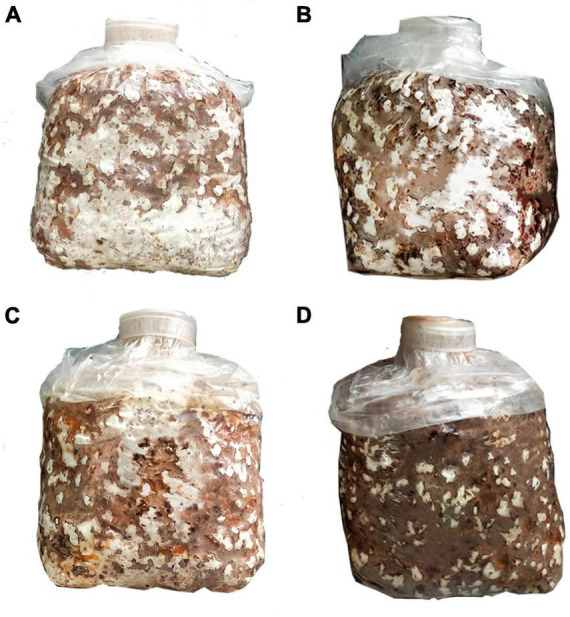
*Lentinula edodes* grown in panel **(A)** the control treatment and treatments supplemented with panel **(B)** 1%, **(C)** 3% and **(D)** 5% hydrated lime.

With the increase in the content of hydrated lime, the time taken for mycelium to change from white to brown gradually decreased, suggesting that the addition of hydrated lime to the medium accelerated the formation of the brown film. The areas of brown film were bigger in the hydrated lime containing treatments T1, T2 and T3 than in the control (CK) (Figure 1). In addition, the polysaccharide contents of the fruiting bodies were higher in the hydrated lime containing treatments than in the control (*P* < 0.05); the contents were 3.5, 4.4, 5.3, and 4.5% in CK, T1, T2, and T3, respectively (Table 1).

**TABLE 1:** The crude polysaccharide contents of *Lentinula edodes* fruiting bodies and enzyme activities in *L. edodes* brown film.

		CK	T1	T2	T3
Crude polysaccharides (%) Enzyme activity (U ml^–1^)	Lignin peroxidase	3.48 ± 0.22*d* 65.24 ± 4.36*b*	4.42 ± 0.51*c* 69.41 ± 0.59*ab*	5.27 ± 0.32*a* 74.86 ± 0.22*a*	4.45 ± 1.11*b* 71.07 ± 0.33*a*
	Manganese peroxidase	8.29 ± 0.03*d*	10.47 ± 0.19*b*	9.21 ± 0.24*c*	14.13 ± 0.18*a*
	Hemicellulase	47.29 ± 0.44*a*	46.15 ± 0.48*a*	43.60 ± 0.57*b*	39.95 ± 0.72*c*
	Cellulase	160.64 ± 0.73*a*	155.39 ± 0.32*b*	156.20 ± 1.36*b*	151.14 ± 1.11*c*
	Laccase	121.40 ± 0.99*d*	131.50 ± 0.75*c*	151.60 ± 0.91*a*	140.53 ± 1.39*b*

Values are mean ± standard deviation (*n* = 3). Different superscript letters on a row indicate statistically significant differences at *P* < 0.05. CK, control treatment; T1, 1% hydrated lime; T2, 3% hydrated lime; T3, 5% hydrated lime.

The activities of laccase and manganese peroxidase (MnP) were higher and that of cellulase was lower in all the hydrated lime treatments than in the control (*P* < 0.05); laccase and MnP activities were above 130 and 9 U ml^–1^, respectively, and cellulase activity below 156 ml^–1^ in the hydrated lime treatments (Table 1). The activity of lignin peroxidase (LiP) was higher and that of hemicellulase was lower in T2 and T3 than in the control (*P* < 0.05); LiP and hemicellulase activities were above 70 ml^–1^ and below 44 ml^–1^, respectively, in T2 and T3 (Table 1).

### 3.2 Sequencing and gene annotation

After filtering, we obtained from 43 million to 45 million reads per treatment (Table 2 and Supplementary Tables 2, 3). Approximately 85% of the reads were mapped, most of them uniquely, onto the *L. edodes* genome (Supplementary Table 4). Approximately 83% of the genome-mapped reads were mapped to gene regions, out of which more than 96% were mapped onto exons (Supplementary Table 5). In total, 9,913 genes were recovered.

**TABLE 2:** Numbers of bases and reads in the transcriptomes of the brown film of *L. edodes*.

	CK	T1	T2	T3
Bases	7414279700	7205274200	7219885300	7055507500
Clean reads	45290330	44044069	44212468	43103647
Mapped reads	39854460	37601591	38848312	37711518
Mapped ratio	88.00%	85.37%	87.87%	87.49%

CK, control treatment; T1, 1% hydrated lime; T2, 3% hydrated lime; T3, 5% hydrated lime.

**FIGURE 2:**
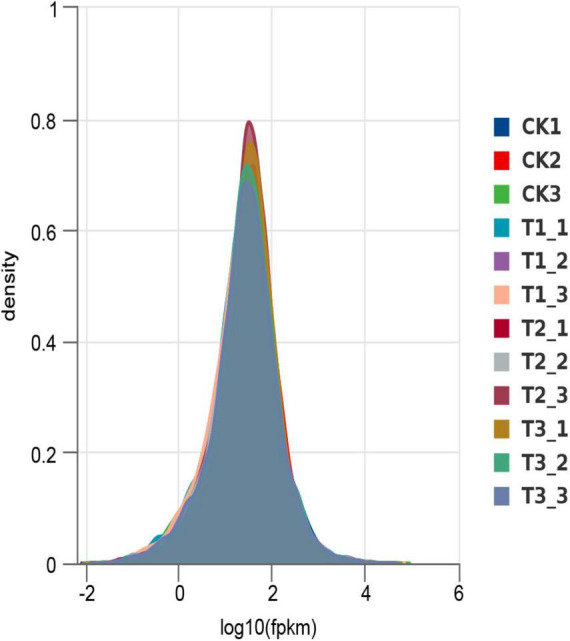
FPKM density distribution.

Based on the fragments per kilobase per million mapped reads (FPKM), the gene expression levels in both the CK, T1, T2, and T3 treatments were similar (Figure 2), implying that the data was amenable to differential expression analysis.

In the *L. edodes* transcriptome, mRNA of 583 genes encoding CAZymes were identified (Supplementary Table 9). Out of the CAZymes genes, 282 were classified into glycoside hydrolases (GHs), 118 into glycosyltransferases (GTs), 99 into auxiliary activities (AAs), 38 into carbohydrate esterases (CEs), 36 into carbohydrate-binding modules (CBMs), and 10 into polysaccharide lyases (PLs) (Supplementary Table 6).

### 3.3 Differentially expressed genes (DEGs)

**FIGURE 3:**
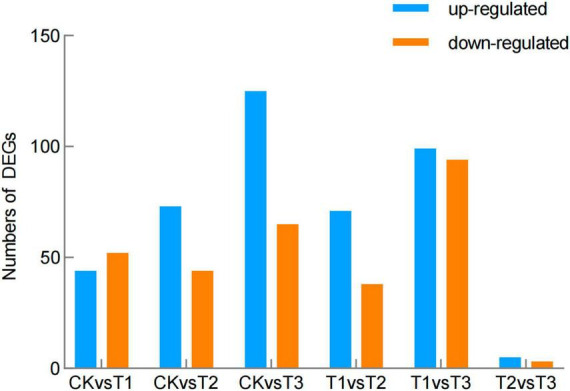
Differentially expressed genes (DEGs) in the brown film of *L. edodes*. CK, control treatment; T1, 1% hydrated lime; T2, 3% hydrated lime; T3, 5% hydrated lime.

Compared to the control, 47 genes were up-regulated and 52 genes down-regulated in T1; 73 genes were up-regulated and 44 were down-regulated in T2; and 125 genes were up-regulated and 65 genes were down-regulated in T3 (Figure 3 and Supplementary Table 7). Compared to T3, 99 genes were up-regulated and 94 down-regulated in T1, and five genes were up-regulated and three genes were down-regulated in T2 (Figure 3 and Supplementary Table 7).

**FIGURE 4:**
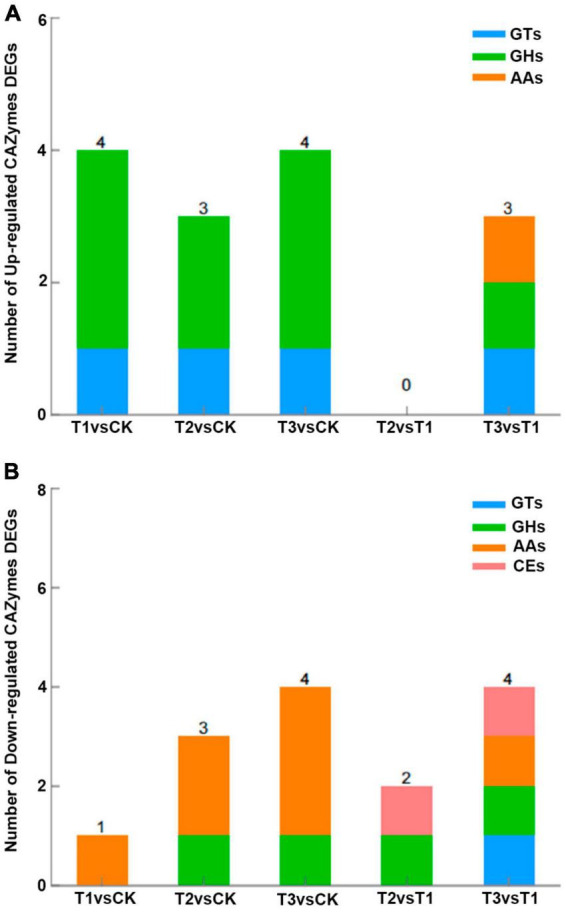
**(A)** Up-regulated and **(B)** down-regulated genes per CAZymes category in *L. edodes*. CK, control treatment; T1, 1% hydrated lime; T2, 3% hydrated lime; T3, 5% hydrated lime. AAs, auxiliary activities; CEs, carbohydrate esterases; GHs, glycosyl hydrolases; GTs, glycosyl transferases.

In the *L. edodes* transcriptome, many genes involved in brown film formation were differentially expressed. Compared with the control, one up- and four down-regulated, three down-regulated, and one down-regulated cytochrome P450 genes, and three up-regulated, two up- and one down-regulated, and three up- and one down-regulated glycoside hydrolase genes were identified in T1, T2, and T3, respectively; hydrophobin 2 gene was down-regulated in T1, T2, and T3 (Table 3). Compared with the control, four CAZymes genes were up-regulated and one down-regulated in T1, three were up-regulated and three down-regulated in T2, and four were up-regulated and four down-regulated in T3 (Figure 4). Eight of the up-regulated genes were GHs and three were GTs, and six of the down-regulated genes were AAs and two were GHs. Compared with T1, three CAZymes genes were up-regulated in T3, and two and four were down-regulated in T2 and T3, respectively (Figure 4).

**TABLE 3:** Differentially expressed genes associated with brown film formation in *L. edodes*.

	Gene ID	Gene annotation	log2FoldChange	*P*-value
T1vsCK	*LENED_001276*	Cytochrome P450 monooxygenase	−1.98	0.00*
	*LENED_000777*	Cytochrome P450 monooxygenase	−1.31	0.00*
	*LENED_008571*	Cytochrome P450 monooxygenase	−1.55	0.04
	*LENED_006243*	Cytochrome P450	−1.97	0.03
	*LENED_012754*	Cytochrome P450 monooxygenase	1.16	0.04
	*LENED_003059*	Glycoside hydrolase family 152 protein	1.06	0.00*
	*LENED_009936*	Glycoside hydrolase family 18 protein	1.07	0.00*
	*LENED_002015*	Glycoside hydrolase family 16 protein	1.15	0.03
	*LENED_002150*	Hydrophobin 2	−1.52	0.00*
T2vsCK	*LENED_006924*	Cytochrome P450 monooxygenase	−1.33	0.00*
	*LENED_001276*	Cytochrome P450 monooxygenase	−2.03	0.00*
	*LENED_002328*	Cytochrome P450	−1.02	0.04
	*LENED_001677*	Glycoside hydrolase family 17 protein	1.85	0.00*
	*LENED_007456*	Glycoside hydrolase family 15 protein	1.18	0.01
	*LENED_012114*	Glycoside hydrolase family 18 protein	−1.19	0.01
	*LENED_002150*	Hydrophobin 2	−2.24	0.00*
T3vsCK	*LENED_001276*	Cytochrome P450 monooxygenase	−1.53	0.01
	*LENED_001677*	Glycoside hydrolase family 17 protein	2.65	0.00*
	*LENED_007456*	Glycoside hydrolase family 15 protein	1.12	0.00*
	*LENED_006657*	Glycoside hydrolase family 16 protein	1.39	0.04
	*LENED_001185*	Glycoside hydrolase family 71 protein	−1.51	0.01
	*LENED_002150*	Hydrophobin 2	−3.16	0.00*

*P-value lower than 0.01. CK, control treatment; T1, 1% hydrated lime; T2, 3% hydrated lime; T3, 5% hydrated lime.

### 3.4 Enrichment analysis of DEGs

**FIGURE 5:**
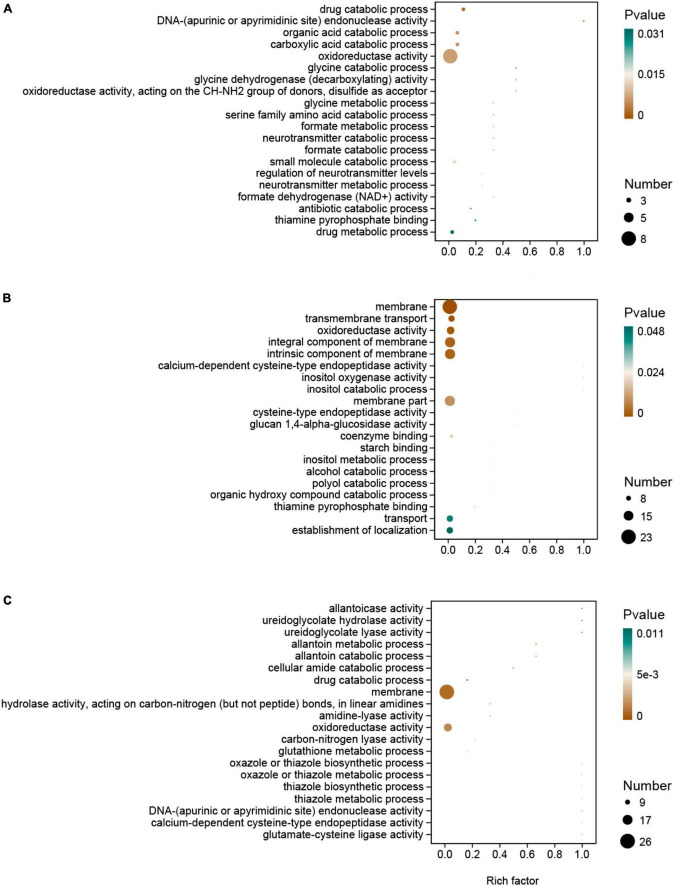
Gene ontology (GO) enrichment of the differentially expressed genes in *L. edodes* in the lime treatments, as compared to the control treatment. **(A)** T1, 1% hydrated lime; **(B)** T2, 3% hydrated lime; **(C)** T3, 5% hydrated lime.

In the GO enrichment analysis, DEGs were enriched in the oxidoreductase activity category in all the hydrated lime treatments compared to the control. In this pathway, genes *LENED_001276*, coding for Cytochrome P450 monooxygenase, and *LENED_008371*, coding for dehydrogenase xptC, were co-down-regulated in all the hydrated lime treatments (Figure 5 and Supplementary Table 8). In the KEGG pathway enrichment analysis, compared with the control, the DEGs in T1 were enriched in glyoxylate and dicarboxylate metabolism and methane metabolism pathways; in T2, DEGs were enriched in MAPK signaling pathway - yeast and ascorbate and aldarate metabolism pathways; and in T3, DEGs were enriched in arachidonic acid metabolism and purine metabolism pathways (*P* < *0.05*) (Table 4 and Supplementary Table 9). Compared with T1, the DEGs in both T2 and T3 were enriched in histidine metabolism pathway. The DEGs between T2 and T3 were enriched in ribosome (*P* < *0.05*) (Table 4 and Supplementary Table 9). Therefore, based on the KEGG pathway enrichment analysis, adding hydrated lime in the growth substrate had influenced the amino acid, carbohydrate and lipid metabolism related pathways (*P* < *0.05*).

**TABLE 4:** The KEGG pathways with highest number of differentially expressed genes in *L. edodes*.

	Pathway	Number of DEGs	*P*-value
T1vsCK	Glyoxylate and dicarboxylate metabolism	2	0.00*
	Methane metabolism	1	0.03
T2vsCK	MAPK signaling pathway - yeast	2	0.02
	Ascorbate and aldarate metabolism	1	0.03
T3vsCK	Arachidonic acid metabolism	1	0.04
	Purine metabolism	2	0.04
T2vsT1	Histidine metabolism	2	0.00*
	Taurine and hypotaurine metabolism	2	0.02
T3vsT1	Histidine metabolism	2	0.00*
	Arachidonic acid metabolism	1	0.01
T3vsT2	Ribosome	1	0.05

*P-value lower than 0.01. CK, control treatment; T1, 1% hydrated lime; T2, 3% hydrated lime; T3, 5% hydrated lime.

### 3.5 qRT-PCR Validation of DEGs

**FIGURE 6:**
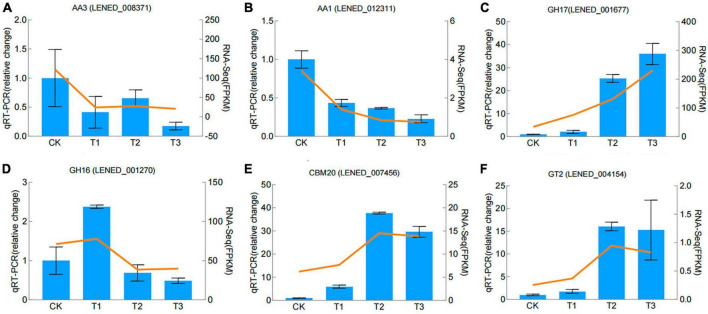
Validation of RNA–seq data by qRT–PCR. Blue color bars represent the relative expression levels determined by qRT–PCR. Orange lines indicate the log2 fold change based on the read count values of the RNA–seq analysis. Error bars indicate standard errors of the means (*n* = 3).

The qRT-PCR gene expression levels of six CAZymes genes, chosen based on their biological roles in GO and KEGG analysis and the differences in gene expression between samples were in line with the differential expression detected in the RNA-Seq analysis (Figure 6).

## 4 Discussion

Many mushrooms favor near to neutral or lightly basic pH for their growth. In addition, pH is an important factor for the quality of the fruiting bodies of *L. edodes*. The pH of the mushroom cultivation substrates is usually adjusted with hydrated lime that contributes to the growth of mycelia and yield of fruiting bodies (Khan et al., 2013). We applied transcriptomics to elucidate the molecular mechanisms behind the effects of hydrated lime on the growth and development of *L. edodes*.

The formation of brown film, a unique development stage in the cultivation of *L. edodes*, affects its yield, quality and production cycle. The rate of brown film formation was faster in the hydrated lime containing treatments than in the control, and the areas of brown film were larger with higher hydrated lime contents. The results suggested that adding hydrated lime to the culture medium may promote the formation of brown film of *L. edodes*, which may shorten the production cycle. The expression of glycoside hydrolase, cytochrome P450 (Du et al., 2019), and hydrophobin (Yoo et al., 2019) related genes are closely related to brown film formation. In the growth of *L. edodes*, glycoside hydrolases play important roles in hydrolyzing lignocellulose in culture medium and providing sufficient nutrition (Yuan et al., 2019; Huang et al., 2020). Cytochrome P450 contributes to vital processes such as carbon source assimilation and biosynthesis of structural components of living organisms (Werck-Reichhart and Feyereisen, 2000). The surface hydrophobicity of different structures is affected by hydrophobins, playing an important role in producing large and complex developmental structures in basidiomycetes (De Groot et al., 1999). In previous studies, glycoside hydrolase, cytochrome P450, and hydrophobin genes were differentially expressed during the brown film formation stage of *L. edodes* (Tang et al., 2013; Song et al., 2018). In line with that, our results indicated that the addition of hydrated lime in the growth substrate may affect the brown film formation of *L. edodes* by affecting the expression of glycoside hydrolase, cytochrome P450 and hydrophobic protein. Since glycoside hydrolase, cytochrome P450, and hydrophobin were affected by pH (Lee et al., 2014; Tucker et al., 2015; Li et al., 2018), their differential expression may have resulted from the effect of hydrated lime on pH.

The polysaccharide contents in *L. edodes* were higher in the hydrated lime treatments than in the control, indicating that an appropriate amount of hydrated lime in the growth substrate could promote polysaccharide synthesis in *L. edodes*. The synthesis of polysaccharides in *L. edodes* is closely related to pentose and glucuronate interconversions and starch and sucrose metabolism pathways (Li et al., 2021). In our study, two genes, NADP-dependent alcohol dehydrogenase and glycoside hydrolase 15 (GH15), were up-regulated in these pathways. The degradation of lignocellulose by white-rot fungi generates toxic compounds that can be detoxified by yeast and *Pleurotus ostreatus*, a white-rot fungus, alcohol dehydrogenases (Liu, 2011; Feldman et al., 2015). The up-regulation of NADP-dependent alcohol dehydrogenase may have resulted as a response to the increased lignocellulose degradation and the accompanying generation of toxic compounds in the hydrated lime treatment. GH15 encodes a glucoamylase that catalyzes the release of β-D-glucose from the no-reducing ends of starch (Sauer et al., 2000). Possibly, the upregulation of GH15 in the hydrated lime treatments was connected to enhanced degradation of starch in the wheat bran part of the substrate, providing glucose for polysaccharide synthesis.

The assembly and degradation of glycans and glycoconjugates is catalyzed by carbohydrate-active enzymes (CAZymes) that play a significant biological role in carbohydrate metabolism (Cantarel et al., 2009; Garron and Henrissat, 2019). In this study, the differentially expressed CAZymes genes included genes in the glycosyl hydrolase (GH), glycosyl transferase (GT), auxiliary activity (AA) and carbohydrate esterase (CE) CAZymes families. The CAZymes genes up-regulated in the hydrated lime treatments were mostly GHs that have been hypothesized to mediate hyphal growth (Vincent et al., 2012), suggesting that GHs are important in mediating the growth of *L. edodes* as well. GHs degrade carbohydrates and glycosidic bonds between carbohydrates and non-carbohydrates (Ma et al., 2020). Therefore, GHs play an important role in the degradation of lignocellulose. The up-regulated GT genes were in the GT1 family that glycosylate natural products, e.g., flavonoids, glycolipids and macrolides (Zhang et al., 2020; Mendoza and Jaña, 2021), and in GT2, encoding a chitin synthase. Chitin, a structural polysaccharide in fungal cell wall, can improve the nutritional value of mushrooms (Vetter, 2007). Thus, hydrated lime may have induced the synthesis of GHs and enzymes in the GT1 and GT2 families, resulting in more substrate for and higher activity in glycosylation, thereby possibly strengthening mycelial cell walls. In addition, *L. edodes* can effectively degrade lignin and cellulose in the growth substrate by secreting large amounts of laccase, lignin peroxidase (LiP) and manganese peroxidase (MnP) (Chen et al., 2016; Zhang et al., 2022), all of which belong to oxidoreductases. The increased activity of these enzymes in the hydrated lime treatments plausibly improved the ability to degrade lignocellulose in the cultivation substrate. However, the CAZymes genes down-regulated in the hydrated lime treatments were mostly in the AA family, encoding enzymes that assist the CE and GH enzymes to access the plant cell wall carbohydrates. The AA1_3 gene, down-regulated in T2 and T3, encodes a laccase-like multicopper oxidase (LMCO) that is considered a key component in the enzyme mixture related to cellulose degradation (Levasseur et al., 2013; Gaber et al., 2020). In line with the down-regulation of AA1_3, cellulase and hemicellulase activities were lower in the hydrated lime treatments than in the control, suggesting that at the brown film formation stage the two enzymes were limited. Thus, despite the putatively greater lignocellulolytic activity, the ability of *L. edodes* to degrade cellulose was lower in the hydrated lime treatments than in the control. The AA3 gene that was down-regulated in all hydrated lime treatments belongs to the AA3_2 aryl/alcohol oxidase subfamily. Similarly, AA3_2 genes were down-regulated in *Laetiporus sulphureus* grown on a substrate where the main carbon source was lignin (de Figueiredo et al., 2021). The results suggest a caveat in lignocellulose degradation. As noted earlier, the toxic components resulting from the degradation of the lignin part interfere with the degradation of cellulose and hemicellulose(Liu, 2011; Feldman et al., 2015); thus, enhanced lignin degradation may have resulted in lower cellulose and hemicellulose degradation rate.

In the GO enrichment analysis, the DEGs were enriched in oxidoreductase activity in all the hydrated lime treatments; oxidoreductases are essential for lignocellulose degradation in basidiomycetes (Mattila et al., 2022) such as *L. edodes*. Together with the increased activity of laccase and peroxidases, it showed that an appropriate amount of hydrated lime in the growth substrate promotes the ability of *L. edodes* mycelium to utilize lignin. The down-regulated gene LENED_008371 codes for dehydrogenase xptC, an enzyme in the fungal prenyl xanthone synthesis pathway (Sützl et al., 2019). The specific function of this gene in the metabolism of *L. edodes* needs to be further clarified. In the KEGG enrichment analysis, compared with the control, the DEGs in T1 were enriched in glyoxylate and dicarboxylate metabolism and methane metabolism pathways, which may be related to the formation of the primordium and spores of edible fungi (Lu et al., 2020; Cai et al., 2022); in T2, DEGs were enriched in MAPK signaling pathway - yeast and ascorbate and aldarate metabolism pathways, which were associated with cell wall integrity maintenance and fruiting body development (Yang et al., 2021); and in T3, DEGs were enriched in arachidonic acid metabolism pathway, possibly providing a source for lipid-derived radicals for lignin degradation (Kapich et al., 1999), and purine metabolism pathway that is associated with the umami taste (Xia et al., 2023), suggesting that hydrated lime treatment may affect the taste of *L. edodes*. The results of enrichment analysis suggested that hydrated lime in the culture substrate may be beneficial for the lignocellulolytic capacity of *L. edodes* and promote the development of fruiting bodies.

In summary, the results suggested that adding a proper amount of hydrated lime in the growth medium promoted the formation of brown film. The crude polysaccharide contents of the fruiting bodies were higher in the hydrated lime treatments. Hydrated lime also promoted the lignocellulolytic capacity of *L. edodes* by enhancing activities of enzymes, thus providing more nutrients for *L. edodes* growth and development. Most of the DEGs were in carbohydrate metabolism related pathways. Among the 583 CAZymes genes, most of the up-regulated genes in the hydrated lime treatments were in the GH family, indicating that these genes were associated with the improved growth and development of *L. edodes*. The exact roles of these genes in *L. edodes* growth and development need further clarification.

## Data availability statement

The datasets presented in this study can be found in online repositories. The names of the repository/repositories and accession number(s) can be found below: https://www.ncbi.nlm.nih.gov/, PRJNA979791.

## Author contributions

YL: Conceptualization; Investigation; Writing – original draft, Writing – review and editing. HW: Writing – original draft. YZ: Investigation; Writing – review and editing. QX: Writing – review and editing. QC: Writing – review and editing. XY: Writing – review and editing. LZ: Writing – review and editing. WP: Writing – review and editing. PP: Writing – review and editing. YG: Conceptualization; Writing – review and editing.

## Footnotes

1
http://www.cazy.org
