# Supplementary material for: Hydrated lime promoted the polysaccharide content and affected the transcriptomes of Lentinula edodes during brown film formation
Source: Front Microbiol. 2023 Dec 4;14:1290180. doi: 10.3389/fmicb.2023.1290180 (PMC10726012; doi:10.3389/fmicb.2023.1290180)
Supplement: Supplementary file 1 [file Table_1.DOCX]

**Supplementary materials**

**Hydrated lime promoted the polysaccharide content and affected the transcriptomes of *Lentinula edodes* during brown film formation**

Yan Li^1†^, Ying Zhang^1†^, Hongcheng Wang^1†^, Quanju Xiang^1^, Qiang Chen^1^, Xiumei Yu^1^, Lingzi Zhang^1^, Weihong Peng^1^, Lujun Zhang^1^, Petri Penttinen^1*^ and Yunfu Gu^1*^

^1^Department of Microbiology, College of Resource , Sichuan Agricultural University, Chengdu 611130, China

***Corresponding author**

1. mail: [guyf@sicau.edu.cn](mailto:guyf@sicau.edu.cn)

[petri.penttinen@helsinki.fi](mailto:petri.penttinen@helsinki.fi)

Table S1. the sequences of seven CAZymes genes primer.

| **Target genes** | **Primer sequences** |
| --- | --- |
| LENED_008371 | Forward: 5'AACAGATGAGGAAGTTGGTCAATGG3' |
|  | Reverse: 5'ACGACACCGCCGAGTTCTC3' |
| LENED_012311 | Forward: 5'ATCATGCGATTCTCTACTGCCAAC3' |
|  | Reverse: 5'GACCGTTTCCCGAGTTTCATCC3' |
| LENED_001677 | Forward: 5'ACGAGGAGAATTTCAAGCAACAAAC3' |
|  | Reverse: 5'GCAACACCGACGCCTATCAG3' |
| LENED_001270 | Forward: 5'ACGCTTCATACCAGTCCAGATTG3' |
|  | Reverse: 5'GACACTCCACATCCCGCATTG3' |
| LENED_007456 | Forward: 5'CCTCCGCTCTGACTGCCTTC3' |
|  | Reverse: 5'ACCTGCTCCTCCTCCTCCTC3' |
| LENED_004154 | Forward: 5'TCCCTTCCCGAGTTCATTAGCC3' |
|  | Reverse: 5'ACACAACAGCACAACCTTCCTC3' |
| GAPDH | Forward: 5'GCAAGGATGCCCCAATGT3' |
|  | Reverse: 5'AGCAAGACAGTTGGTTGTGCAG3' |

Table S2. Summary of the sequencing and assembly.

| **Sample** | **Library Type** | **Reads Length(bp)** | **Raw Reads** | **Raw Data(bp)** | **Q20(%)** |
| --- | --- | --- | --- | --- | --- |
| CK-1 | Paired-End | 150 | 47863888 | 7179583200 | 97.97 |
| CK-2 | Paired-End | 150 | 48353850 | 7253077500 | 98.10 |
| CK-3 | Paired-End | 150 | 52067856 | 7810178400 | 97.44 |
| T1-1 | Paired-End | 150 | 44080830 | 6612124500 | 97.82 |
| T1-2 | Paired-End | 150 | 44061414 | 6609212100 | 97.94 |
| T1-3 | Paired-End | 150 | 55963240 | 8394486000 | 98.00 |
| T2-1 | Paired-End | 150 | 46960048 | 7044007200 | 97.87 |
| T2-2 | Paired-End | 150 | 44237852 | 6635677800 | 98.07 |
| T2-3 | Paired-End | 150 | 53199806 | 7979970900 | 98.04 |
| T3-1 | Paired-End | 150 | 46047970 | 6907195500 | 97.96 |
| T3-2 | Paired-End | 150 | 45991432 | 6898714800 | 98.03 |
| T3-3 | Paired-End | 150 | 49070748 | 7360612200 | 97.93 |

Table S3. Summary of the sequencing data filtered by Q20.

| **Sample** | **Library Type** | **Reads** | **Data(bp)** | **Reads(%)** | **Data(%)** |
| --- | --- | --- | --- | --- | --- |
| CK-1 | Paired-End | 44039142 | 6605871300 | 92.00 | 92.00 |
| CK-2 | Paired-End | 44476328 | 6671449200 | 91.98 | 91.98 |
| CK-3 | Paired-End | 47355520 | 7103328000 | 90.94 | 90.94 |
| T1-1 | Paired-End | 40622280 | 6093342000 | 92.15 | 92.15 |
| T1-2 | Paired-End | 40494552 | 6074182800 | 91.90 | 91.90 |
| T1-3 | Paired-End | 51015374 | 7652306100 | 91.15 | 91.15 |
| T2-1 | Paired-End | 43132866 | 6469929900 | 91.85 | 91.85 |
| T2-2 | Paired-End | 40515266 | 6077289900 | 91.58 | 91.58 |
| T2-3 | Paired-End | 48989272 | 7348390800 | 92.08 | 92.08 |
| T3-1 | Paired-End | 42154044 | 6323106600 | 91.54 | 91.54 |
| T3-2 | Paired-End | 42269406 | 6340410900 | 91.90 | 91.90 |
| T3-3 | Paired-End | 44887492 | 6733123800 | 91.47 | 91.47 |

Table S4. Mapped results of the RNA sequencing data.

| **Sample** | **Useful Reads** | **Map Events Count** | **Total Mapped** | | **Multiple Mapped** | | **Uniquely Mapped** | |
| --- | --- | --- | --- | --- | --- | --- | --- | --- |
|  |  |  | **Reads** | **%** | **Reads** | **%** | **Reads** | **%** |
| CK-1 | 44,039,142 | 38,463,496 | 38,973,403 | 88.50 | 509,907 | 1.31 | 38,463,496 | 98.69 |
| CK-2 | 44,476,328 | 38,646,334 | 39,156,606 | 88.04 | 510,272 | 1.30 | 38,646,334 | 98.70 |
| CK-3 | 47,355,520 | 40,897,620 | 41,433,371 | 87.49 | 535,751 | 1.29 | 40,897,620 | 98.71 |
| T1-1 | 40,622,280 | 33,748,320 | 34,178,501 | 84.14 | 430,181 | 1.26 | 33,748,320 | 98.74 |
| T1-2 | 40,494,552 | 34,413,114 | 34,834,204 | 86.02 | 421,090 | 1.21 | 34,413,114 | 98.79 |
| T1-3 | 51,015,374 | 43,240,877 | 43,792,069 | 85.84 | 551,192 | 1.26 | 43,240,877 | 98.74 |
| T2-1 | 43,132,866 | 37,292,675 | 37,770,528 | 87.57 | 477,853 | 1.27 | 37,29,2675 | 98.73 |
| T2-2 | 40,515,266 | 35,304,506 | 35,763,063 | 88.27 | 458,557 | 1.28 | 35,30,4506 | 98.72 |
| T2-3 | 48,989,272 | 42,432,331 | 43011346 | 87.80 | 579,015 | 1.35 | 42,432,331 | 98.65 |
| T3-1 | 42,154,044 | 36,381,404 | 36,846,984 | 87.41 | 465,580 | 1.26 | 36,381,404 | 98.74 |
| T3-2 | 42,269,406 | 36,393,170 | 36,866,385 | 87.22 | 473,215 | 1.28 | 36,393,170 | 98.72 |
| T3-3 | 44,887,492 | 38,923,381 | 39,421,185 | 87.82 | 497,804 | 1.26 | 38,923,381 | 98.74 |

Table S5. Detailed analysis of gene map counts.

| **Sample** | **Map Events Count** | **Mapped to Gene** | | **Mapped to InterGene** | | **Mapped to Exon** | |
| --- | --- | --- | --- | --- | --- | --- | --- |
|  |  | **count** | **%** | **count** | **%** | **count** | **%** |
| CK-1 | 38,463,496 | 32282724 | 83.93 | 6,180,772 | 16.07 | 31,329,192 | 97.05 |
| CK-2 | 38,646,334 | 33397078 | 86.42 | 5,249,256 | 13.58 | 32,382,614 | 96.96 |
| CK-3 | 40,897,620 | 35630153 | 87.12 | 5,267,467 | 12.88 | 34,637,063 | 97.21 |
| T1-1 | 33,748,320 | 28916866 | 85.68 | 4,831,454 | 14.32 | 28,109,244 | 97.21 |
| T1-2 | 34,413,114 | 29735386 | 86.41 | 4,677,728 | 13.59 | 28,876,330 | 97.11 |
| T1-3 | 43,240,877 | 37600983 | 86.96 | 5,639,894 | 13.04 | 36,551,436 | 97.21 |
| T2-1 | 37,292,675 | 32240227 | 86.45 | 5,052,448 | 13.55 | 31,279,200 | 97.02 |
| T2-2 | 35,304,506 | 30532101 | 86.48 | 4,772,405 | 13.52 | 29,647,532 | 97.10 |
| T2-3 | 42,432,331 | 36267620 | 85.47 | 6,164,711 | 14.53 | 35,087,358 | 96.75 |
| T3-1 | 36,381,404 | 31193841 | 85.74 | 5,187,563 | 14.26 | 30,195,558 | 96.80 |
| T3-2 | 36,393,170 | 31137934 | 85.56 | 5,255,236 | 14.44 | 30,151,836 | 96.83 |
| T3-3 | 38,923,381 | 33988914 | 87.32 | 4,934,467 | 12.68 | 33,065,687 | 97.28 |
